# Plastid Glycerol-3-phosphate Acyltransferase Enhanced Plant Growth and Prokaryotic Glycerolipid Synthesis in *Brassica napus*

**DOI:** 10.3390/ijms21155325

**Published:** 2020-07-27

**Authors:** Huiling Kang, Chenxi Jia, Nian Liu, Alfatih Alamin Alhussain Aboagla, Wenling Chen, Wei Gong, Shaohua Tang, Yueyun Hong

**Affiliations:** National Key Laboratory of Crop Genetic Improvement, Huazhong Agricultural University, Wuhan 430070, China; 15927316487@163.com (H.K.); jiachenxi0701@163.com (C.J.); galaxy_liu163@163.com (N.L.); alfatih.alamin2015@gmail.com (A.A.A.A.); chenwl828@163.com (W.C.); 13163256438@163.com (W.G.); amy.feather@163.com (S.T.)

**Keywords:** glycerol-3-phosphate acyltransferase, lipid metabolism, prokaryotic glycerolipids, plant growth

## Abstract

Plastid-localized glycerol-3-phosphate acyltransferase (ATS1) catalyzes the first-step reaction in glycerolipid assembly through transferring an acyl moiety to glycerol-3-phosphate (G3P) to generate lysophosphatidic acid (LPA), an intermediate in lipid metabolism. The effect of ATS1 overexpression on glycerolipid metabolism and growth remained to be elucidated in plants, particularly oil crop plants. Here, we found that overexpression of *BnATS1* from *Brassica napus* enhanced plant growth and prokaryotic glycerolipid biosynthesis. BnATS1 is localized in chloroplasts and an in vitro assay showed that BnATS1 had acylation activity toward glycerol 3-phosphate to produce LPA. Lipid profiling showed that overexpression of *BnATS1* led to increases in multiple glycerolipids including phosphatidylglycerol (PG), monogalactosyldiacylglycerol (MGDG), phosphatidylcholine (PC), and phosphatidylinositol (PI), with increased polyunsaturated fatty acids. Moreover, increased MGDG was attributed to the elevation of 34:6- and 34:5-MGDG, which were derived from the prokaryotic pathway. These results suggest that BnATS1 promotes accumulation of polyunsaturated fatty acids in cellular membranes, thus enhances plant growth under low-temperature conditions in *Brassica napus*.

## 1. Introduction

Glycerolipids including phospholipids and galactolipids are essential components of cellular membranes and signal molecules involved in diverse biological processes [1]. Glycerol-3-phosphate acyltransferase (GPAT) catalyzes the first-step reaction in glycerolipid assembly through transferring an acyl moiety to glycerol-3-phosphate (G3P), producing lysophosphatidic acid (LPA), which is further acylated at the sn-2 position of the G3P backbone by lysophosphatidic acid acyltransferase (LPAAT) to generate phosphatidic acid (PA) [2,3,4]. PA is an important intermediate in glycerolipid metabolism. PA is activated by cytidinediphosphate diacylglycerol synthase (CDS) to produce cytidinediphosphate diacylglycerol (CDP-DAG), which is a precursor for phosphatidylglycerol (PG) and phosphatidylinositol (PI) [5,6,7]. Alternatively, PA is dephosphorylated by PA phosphohydrolase (PAH) or lipid phosphate phosphatase (LPP) to produce diacylglycerol (DAG) [8,9], which subsequently reacts with cytidinediphosphate-choline (CDP-choline) and CDP-ethanolamine to produce phosphatidylcholine (PC) and phosphatidylethanolamine (PE), respectively [1,10,11]. DAG is also a substrate for galacotolipids such as monogalactosyldiacylglycerol (MGDG), digalactosyldiacylglycerol (DGDG) [12], and storage lipid triacylglycerol (TAG) as well [13].

In plants, fatty acids are synthesized de novo in chloroplasts, and are either retained in the chloroplasts for membrane lipid assembly via the prokaryotic pathway or exported to the endoplasmic reticulum (ER) for synthesis of membrane lipids and storage TAG via the eukaryotic pathway [1,2] (Supplementary Figure S1). Both PA and its dephosphorylated DAG can be produced via the two pathways [2,14]. The prokaryotic pathway-derived PA and DAG are retained in chloroplasts for the synthesis of PG and galactolipids [1]. The eukaryotic pathway-derived glycerolipids can reenter the chloroplasts, in the form of PA or presumably DAG produced from phospholipids, particularly PC, for the synthesis of thylakoid lipids [8,15,16,17,18,19]. The lipid molecular species derived from the two pathways are distinguishable. Glycerolipids originated from the prokaryotic pathway feature with a 16-carbon (C16) acyl moiety at the sn-2 position of the glycerol backbone, whereas those from the eukaryotic pathway have an 18-carbon (C18) acyl at the sn-2 position due to differing substrate selectivity of acyltransferases between the two pathways in plants [20,21] (Supplementary Figure S1). Thylakoid membrane lipids are derived from the two pathways depending on plant species or tissue.

The Arabidopsis genome contains 10 GPATs, which include a soluble GPAT, namely the plastidic acyl–acyl carrier protein (ACP) GPAT (ATS1) [3,4], and nine membrane-associated GPATs designated GPAT1 to GPAT9 localized to the ER or mitochondria [22,23,24,25,26,27,28]. Most GPAT family members, GPAT1 to GPAT8, catalyze the sn-2 acylation of G3P to produce LPA and are involved in the synthesis of extracellular lipids such as cutin and suberin [22,23,24,25,26,27,28,29,30]. Of these GPATs, GPAT9 is more closely related to animal GPAT3 and is responsible for the synthesis of TAG and phospholipids in the eukaryotic pathway in Arabidopsis [31,32]. ATS1 is localized in chloroplasts involved in the prokaryotic pathway. ATS1 utilizes acyl–acyl carrier protein (acyl-ACP) as a substrate for acylation at the sn-1 position of G3P to produce sn-1 acyl-LPA, which is subsequently acylated by plasidic LPAAT1 (also named as ATS2) to produce PA [3,4,33]. It was reported that ATS1 is essential for PG synthesis in plastids and loss of ATS1 led to a marked reduction in prokaryotic galactolipids in plants [3,4,34]. However, the effect of *ATS1* overexpression on glycerolipid metabolism and growth remained to be elucidated in plants, particularly in crop plants. In this study, we found that overexpression of *Brassica napus ATS1* (*BnATS1*) enhances plant growth and prokaryotic glycerolipid biosynthesis in *Brassica napus.*

## 2. Results

### 2.1. BnATS1 Is Expressed in Various Tissues and BnATS1 Is Localized in Chloroplasts

**Figure 1:**
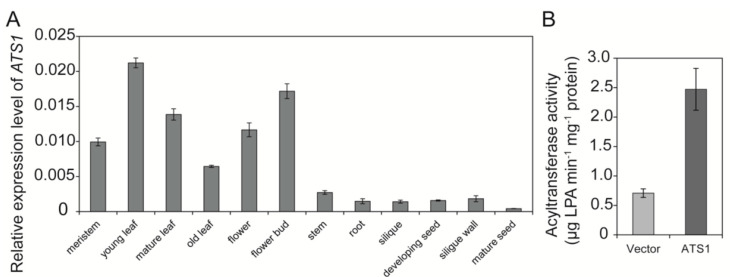
*BnATS1* expression pattern and BnATS1 activity. (**A**) The expression pattern of *BnATS1* in *Brassica napus* detected by RT-qPCR normalized to *BnActin*. Total RNA was extracted from various tissues of cv. Westar. Values are means ± SD (*n* = 3 separate samples). Leaves, roots, meristem, and stems were sampled from 40-day-old plants; flowers and flower buds were sampled from plants at the flowering stage; siliques, 10 days after pollination; developing seeds, 20 days after pollination; developing silique walls, 20 days after pollination; mature seeds, 45 days after pollination. (**B**) BnATS1 catalyzes the acylation of glycerol-3-phosphate using C16:0-CoA as an acyl donor to generate lysophosphatidic acid (LPA) in vitro. Values are means ± SD (*n* = 3 independent experiments). LPA, lysophosphatidic acid.

**Figure 2:**
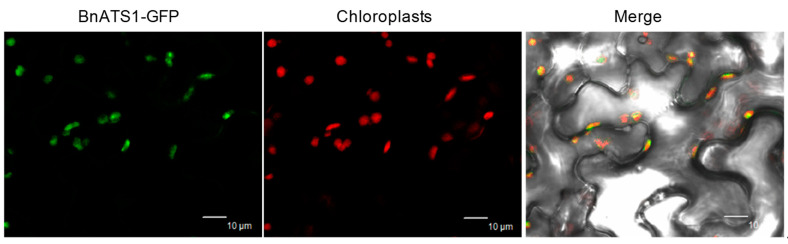
BnATS1 was localized in chloroplasts. BnATS1-GFP was transiently expressed in tobacco leaf cells under the control of the *35S* promoter. Green fluorescent signal of BnATS1-GFP was overlaid with red auto-fluorescence produced by chloroplasts observed using a confocal laser scanning microscope. Bars = 10 μm.

To investigate the temporal and spatial distribution of *BnATS1* mRNA in *Brassica napus*, total RNA was extracted from various tissues and used for analysis by quantitative real-time PCR. *BnATS1* (BnaA08g06960D) is expressed in various tissues including leaves, stems, roots, flowers, flower buds, siliques, developing seeds, developing silique walls, and mature seeds with expression being highest in young leaves and lowest in mature seeds (Figure 1A). The results suggest that *BnATS1* is highly expressed in green tissues, particularly in actively growing leaf tissues. To further explore the subcellular localization and activity of BnATS1, the full-length *BnATS1* coding sequence (CDS) was cloned from cultivar (cv.) Westar (*Brassica napus* L.) by reverse transcription PCR using mRNA from leaves as a template, and the resultant CDS was fused with GFP at the C-terminus and then transiently expressed in the epidermal cells of tobacco leaves by *Agrobacterium* infiltration. Green fluorescent BnATS1-GFP was overlaid with the red fluorescence of chloroplasts (Figure 2), suggesting that BnATS1 is localized to chloroplasts and may be involved in the prokaryotic pathway. To test whether *BnATS1* encodes a GPAT, BnATS1 was expressed in *E. coli* cells in an enzymatic activity assay. The resulting protein was able to catalyze the acylation of G3P using 16:0-CoA as a substrate to produce LPA (Figure 1B).

### 2.2. Overexpression of BnATS1 Enhanced Plant Growth during Vegetative Stage

**Figure 3:**
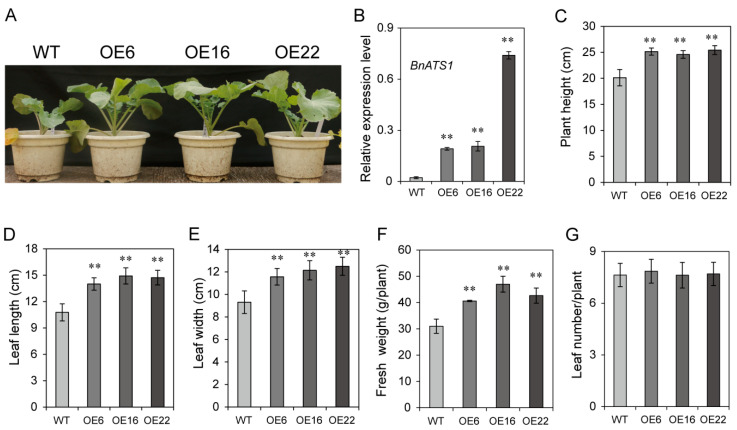
Overexpression of *BnATS1* promoted plant growth at the vegetative stage. (**A**) The growth phenotype. (**B**) *BnATS1* was overexpressed in cv. Westar plants as compared with the wild type (WT) plants detected by RT-qPCR. Total RNA was extracted from leaves of plants at the vegetative stage. *β-Actin* from *Brassica napus* was used as an internal standard. Values are mean ± SD (*n* = 3 separate samples). (**C**) Plant height, (**D**) leaf length, (**E**) leaf width, (**F**) fresh weight of aboveground parts, and (**G**) leaf number of 26-day-old plants under field growth conditions. The leaf size was measured in the top 1st and 2nd leaves fully expanded in 26-day-old plants. The plant height represents the length of aboveground parts. Values are means ± SD (*n* = 5 independent experiments). ** indicates significant difference at *p* < 0.01 compared with the WT based on Student’s *t* test.

*BnATS1* is highly expressed in young leaves, suggesting a role in actively growing tissues. To investigate whether BnATS1 is involved in plant growth, *BnATS1* was overexpressed in cv. Westar (*Brassica napus* L.) plants under the control of the CaMV *35S* promoter. More than 20 independent transgenic lines were obtained and *BnATS1*-overexpression (OE) plants exhibited a similar phenotype with enhanced vegetative growth in the field growth conditions, in which the temperature was approximately 7–10 °C (night)/15–20 °C (day) in fall and 0–3 °C (night)/7–12 °C (day) in winter (Figure 3A). We randomly selected three lines of them for functional characterization in detail. The *BnATS1* transcript level in leaves of OE6, OE16, and OE22 plants was 9- to 33-fold higher than that of the wild type (WT) (Figure 3B). The plant height of *BnATS1*-OE was approximately 30% greater relative to the WT at the vegetative stage (Figure 3C). The leaf length and width in *BnATS1*-OE plants were increased by approximately 34% and 29%, respectively, as compared with the WT plants (Figure 3D,E). The fresh weight of the OE6, OE16, and OE22 lines was increased by 31%, 50%, and 38%, respectively, as compared with the WT at the vegetative stage (Figure 3F). However, the leaf number of *BnATS1*-OE plants was comparable to that of WT plants (Figure 3G). Thus, enhanced fresh weight was primarily due to increased leaf size in *BnATS1*-OE plants.

### 2.3. The Effect of BnATS1 on Phospholipid Metabolism

**Figure 4:**
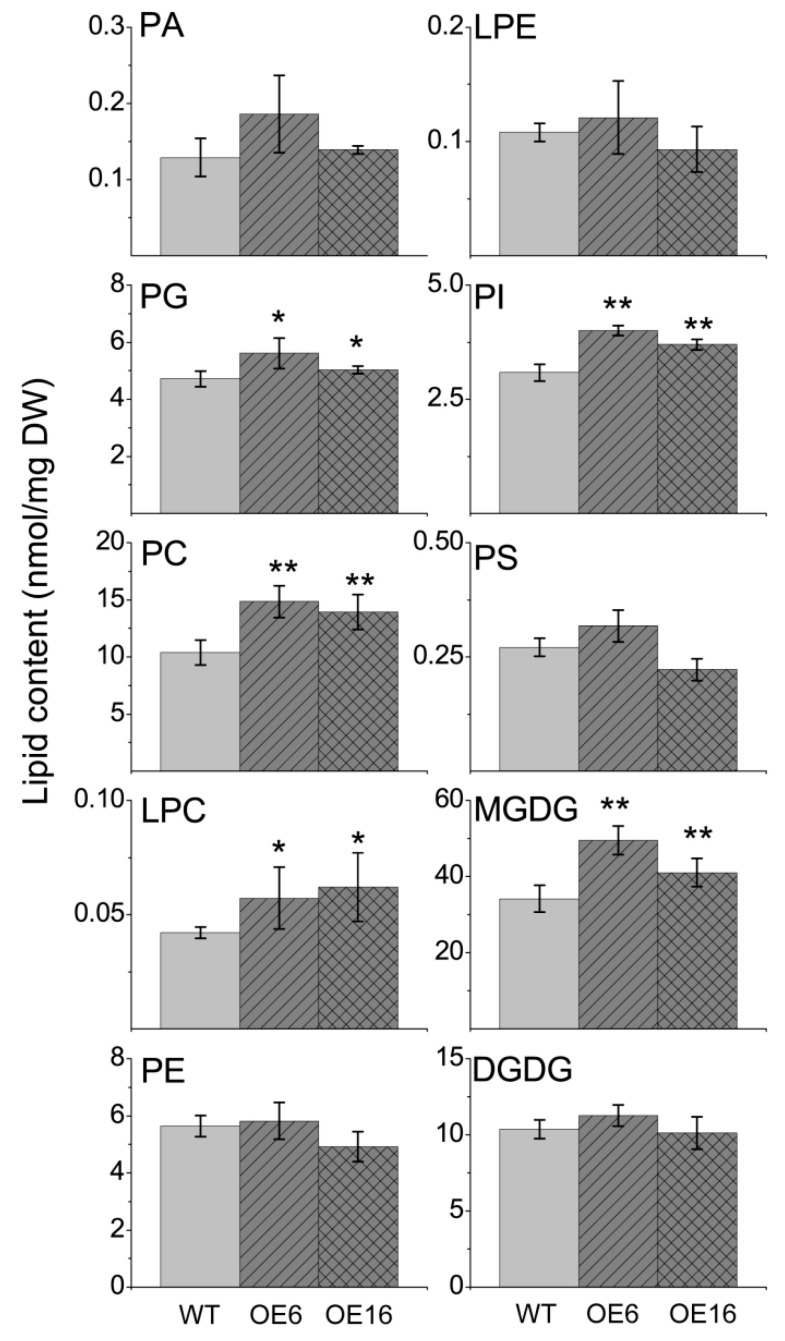
Effect of *BnATS1*-overexpression on membrane lipids. Lipids were extracted from leaves of 40-day-old plants under field growth conditions. Values are means ± SD (*n* = 4 separate samples). * and ** denote significance at *p* < 0.05 and *p* < 0.01, respectively, compared with WT plants based on Student’s *t* test. PA, phosphatidic acid; PG, phosphatidylglycerol; PC, phosphatidylcholine; LPC, lysophosphatidylcholine; PE, phosphatidylethanolamine; LPE, lysophosphatidylethanolamine; PI, phosphatidylinositol; PS, phosphatidylserine; MGDG, monogalactosyldiacylglycerol; DGDG, digalactosyldiacylglycerol.

**Figure 5:**
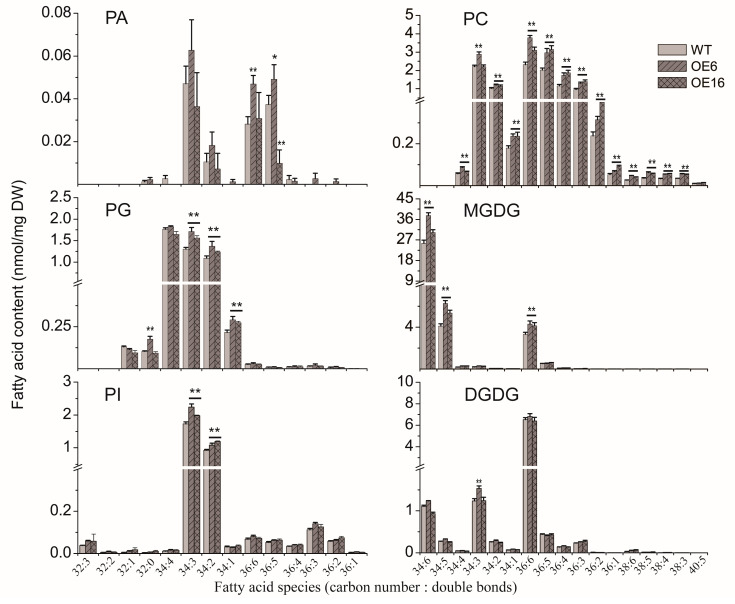
Effect of *BnATS1*-overexpression on membrane lipid composition. Lipids were extracted from leaves of 40-day-old plants under field growth conditions. Fatty acid species are shown as total acyl carbons: total double bonds. Values are means ± SD (*n* = 4 separate samples). * and ** denote significant difference at *p* < 0.05 and *p* < 0.01, respectively, compared with WT plants based on Student’s *t* test.

The results from the subcellular localization and activity assay suggest that BnATS1 catalyzes the first step of glycerolipid assembly to produce LPA in the prokaryotic pathway. To get insight into how BnATS1 affects plant growth, we profiled polar glycerolipid contents and lipid species from leaves of two representative *BnATS1*-OE lines, OE6 and OE16, compared with WT plants at the vegetative growth stage. Lipids from leaves were quantitatively analyzed by electron-spray ionization tandem mass spectrometry (ESI-MS/MS). PA content in leaves was very low at 0.129 nmol/mg DW, representing 0.19% of the total polar glycerolipids examined in WT leaves of cv. Westar (*Brasscia napus*), and total PA content in *BnATS1*-OE leaves was not significantly different from that of WT plants (Figure 4). The molecular species including 34:3-, 34:2-, 36:6-, and 36:5-PA are major constituents of PA. The contents of most PA species in *BnATS1*-OE plants were not significantly different from that of the WT except higher 36:6- and 36:5-PA in OE6 and a lower 34:3-PA in OE16 relative to the WT (Figure 5). The results implicate that PA is rapidly transferred to DAG and CDP-DAG for other glycerolipid synthesis.

PG is the only major phospholipid in thylakoid membranes, which is mainly produced in the prokaryotic pathway in plants [4]. Lipid profiling showed that PG content was 6.8% of the total polar glycerolipids examined in WT leaves of cv. Westar. The major molecular species of PG were 34:4-, 34:3-, and 34:2-PG. Overexpression of *BnATS1* led to an increased PG content compared with the WT (Figure 4). Increased PG in *BnATS1*-OE leaves resulted from increases in 34:3-, 34:2-, and 34:1-PG, which were partially coincided with those major species of PA (Figure 5). The results suggest that increased PG may be derived from PA mediated by *BnATS1* overexpression.

PC is a major phospholipid in cellular membranes and is originally derived from PA via the CDP-choline pathway, and PC can be also produced via the PE methylation pathway [1]. PC content was approximately 15%, whereas other phospholipids such as PE, PI, and phosphatidylserine (PS) were only 8.2%, 4.5%, and 0.4%, respectively, of the total polar glycerolipids examined in WT leaves of cv. Westar (Figure 4). PC in *BnATS1*-OE plants was increased by 34–43%, compared with the WT (Figure 4). LysoPC (LPC) content in *BnATS1*-OE was also higher than that of the WT. Molecular profiling revealed that PC was predominantly composed of 34:3-, 34:2-, 36:6-, 36:5-, 36:4-, and 36:3-PC species. Elevated PC in *BnATS1*-OE leaves can be attributed to increases in almost all PC species, including 34:4-, 34:3-, 34:2-, 34:1-, 36:6-, 36:5-, 36:4-, 36:3-,36:2-, 36:1-, 38:6-, 38:5-, 38:4-, and 38:3-PC (Figure 5). In addition, PI content in *BnATS1*-OE plants was also higher than that of the WT, which was caused by elevations in 34:3- and 34:2-PI (Figure 4 and Figure 5). By comparison, overexpression of *BnATS1* did not lead to changes in PE and PS levels as compared to the WT (Figure 4). The results suggest that increased *BnATS1* expression also promotes PC and PI production with increased polyunsaturated fatty acid species.

### 2.4. Overexpression of BnATS1 Enhanced Prokaryotic Galactolipids

Galactolipids, such as MGDG and DGDG, are mostly abundant in green tissues, particularly in leaves of plants. MGDG and DGDG were 49.6% and 15.1%, respectively, of the total polar glycerolipids examined in WT leaves of cv. Westar (Figure 4). Lipid profiling showed that molecular species of MGDG were distinguishable from those in DGDG. MGDG in leaves were predominantly composed of 34:6-MGDG (18:3/16:3-MGDG), comprising 75% of the total MGDG species, whereas DGDG was mainly composed of 36:6-DGDG (18:3/18:3-DGDG), comprising 63% of the total DGDG species in WT leaves (Figure 5). The results suggest that MGDG is mainly assembled through the prokaryotic pathway in *Brassica napus*. Moreover, overexpression of *BnATS1* led to a significant increase in MGDG. The MGDG contents in OE6 and OE16 leaves were increased by 45% and 20%, respectively, as compared with WT plants. The increased MGDG in *BnATS1*-OE plants was caused predominantly by the elevations in 34:6- 34:5-, and 36:6-MGDG (Figure 5). Although overexpression of *BnATS1* did not affect the total DGDG content, 34:3-DGDG in the OE6 line was higher than that of the WT (Figure 5). These results suggest that enhanced thylakoid lipids in *BnATS1*-OE plants are due to enhanced galactolipid synthesis via the prokaryotic pathway mediated by BnATS1.

### 2.5. Overexpression of BnATS1 Up-Regulated the Expression of Genes Related to Lipid Anabolism

**Figure 6:**
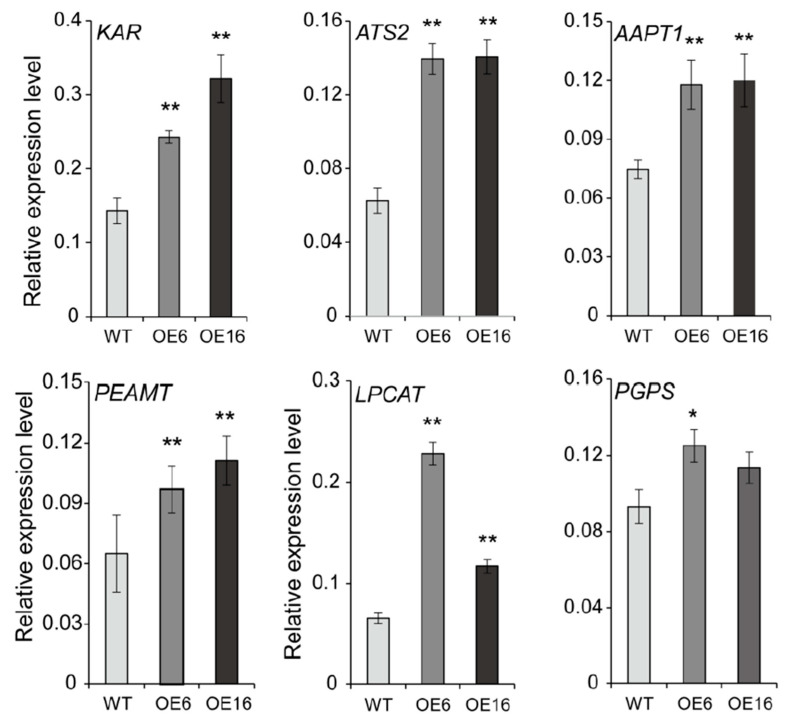
Overexpression of *BnATS1* enhanced expression of genes related to the lipid anabolic process. Total RNA was extracted from leaves of 40-day-old plants under field growth conditions. The relative expression levels of genes were analyzed by real-time PCR normalized to the *β-Actin* expression level. Values are means ± SD (*n* = 3 separate samples). * and ** denote significance at *p* < 0.05 and *p* < 0.01, respectively, compared with WT plants based on Student’s *t* test.

Lipid profiling showed that overexpression of *BnATS1* promoted simultaneously the accumulation of multiple lipids, such as PG, MGDG, PC, and PI. It is possible that overexpression of *BnATS1* may affect the expression of genes involved in different lipid metabolic processes. To test this possibility, RNA was extracted from the leaves of *BnATS1*-OE and WT plants and analyzed by real-time PCR. The β-ketoacyl-ACP reductase (KAR) is required for de novo FA synthesis [35], and the *KAR* expression level in *BnATS1*-OE leaves was two-fold higher than that of the WT (Figure 6). ATS2 catalyzes the sn-2 acylation of the glycerol backbone to produce PA in the prokaryotic pathway following ATS1 action [33], and the *ATS2* transcript level in *BnATS1*-OE leaves was increased two-fold compared with the WT (Figure 6). Moreover, overexpression of *BnATS1* led to up-regulation of genes, such as *PEAMT* (phosphoethanolamine N-methyltransferase) and *AAPT1* (aminoalcoholphosphotransferase 1), involved in PC synthesis from DAG, and *LPCAT* (lysophosphatidylcholine acyltransferase) in PC-acyl editing. In addition, overexpression of *BnATS1* also promoted the expression of *PGPS* (phosphatidylglycerolphosphate synthase) involved in PG synthesis. The *PGPS* expression was significantly up-regulated in OE6 and slightly increased in OE16 as compared with the WT (Figure 6). Taken together, the results suggest that overexpression of *BnATS1* is able to promote synchronously the expression of genes involved in multiple lipid anabolic processes, thus coordinates with other enzymes to enhance glycerolipid synthesis in *Brassica napus*.

## 3. Discussion

In plants, glycerolipids are assembled via two parallel pathways, the plastid-localized prokaryotic pathway and the ER-localized eukaryotic pathway. ATS1 catalyzes a committed step reaction in the prokaryotic pathway. The Arabidopsis mutant *ats1* (also designated *atc1*) with defective ATS1 exhibits a reduction in prokaryotic thylakoid lipids [3,4]. However, the effect of overexpressing *ATS1* on plant growth and lipid metabolism remained to be elucidated. Here, we found that plastid-localized BnATS1 in *Brassica napus* has a positive impact on plant growth, accompanied by increases in multiple glycerolipids including PG, MGDG, PC, and PI.

Our results from *Brassica napus* plants overexpressing *BnATS1* showed that BnATS1 has a promotion on vegetative growth, particularly leaves. Indeed, *BnATS1* was highly expressed in green tissues, being the highest in young leaves, suggesting its role in leaf growth. Our observation showed that BnATS1 is localized in chloroplasts, implicating its involvement in lipid assembly in the prokaryotic pathway. Fatty acids are synthesized de novo in chloroplasts [2], and this plastid-localized BnATS1 may facilitate the access of acyl groups for glycerolipid assembly. It was showed that ATS1 catalyzes the sn-1 acylation of G3P using 18:1-ACP and 16:0-ACP as substrates in *Helianthus annuus* [34]. Our result from the in vitro assay showed that BnATS1 is capable of using 16:0-CoA as a substrate to produce LPA, which is a precursor for PA synthesis through the acylation at the sn-2 of glycerol backbone. PA is an intermediate in glycerolipid metabolism and is rapidly transferred to other lipids [1,2,36]. PA produced in the prokaryotic pathway is responsible for synthesis of PG and galactolipids [15,16], whereas PA produced in the eukaryotic pathway is primarily involved in phospholipid synthesis, and could be transported to chloroplasts for galactolipid synthesis [1,17,18,19]. Our results from the lipid profiling showed that most PA species in *BnATS1*-OE plants were not significantly higher than in the WT. Instead, overexpression of *BnATS1* led to increased PG in leaves. The increased molecular species of PG were mostly similar to those species abundant in PA, implicating that increased PG in *BnATS1*-OE plants may be derived from PA. PG is an essential component of photosynthetic membranes and most PG (approximately 85% of total PG) in plastids is derived from the prokaryotic pathway in Arabidopsis [37]. Increased PG in *BnATS1*-OE plants agrees with a previous report showing that the *ats1* (*act1*) mutant had marked reduction in PG [3].

Galactolipids, such as MGDG and DGDG, are most abundant in land plants, which predominantly present in thylakoid membranes and are essential for photoautotrophic growth [12,38,39]. In Arabidopsis, MGDG in leaves is composed of comparable proportions between eukaryotic species (18:3/18:3-MGDG) and prokaryotic 18:3/16:3-MGDG, whereas DGDG is mostly composed of eukaryotic species (18:3/18:3-DGDG) [37]. Our lipid profiling revealed that MGDG in the leaves of *Brassica napus* was predominantly composed of 34:6-MGDG (18:3/16:3-MGDG) derived from the prokaryotic pathway, whereas DGDG is mainly 36:6-DGDG (18:3/18:3-DGDG) originated from the eukaryotic pathway. Moreover, *BnATS1*-OE plants exhibited an increased MGDG with elevated 34:6- and 34:5-MGDG, as compared with the WT, but the DGDG in *BnATS1*-OE plants is similar to that of the WT. The results suggest BnATS1 contributes to prokaryotic galactolipid accumulation, which agrees with previous reports showing that *ats1* mutant deficiency is in the prokaryotic pathway [3,4]. In addition, *BnATS1*-OE plants had an increased 36:6-MGDG, which may be derived from increased 36:6-PC. PC, a major site of fatty acid desaturation, provides a DAG moiety with polyunsaturated acyl groups for eukaryotic galactolipids [1,2].

Overexpression of *BnATS1* also led to increased PC, which can be attributed to all molecular species of PC examined in leaves, as compared with the WT. PC is a major phospholipid in cellular membranes and an entry point for newly synthesized fatty acids from chloroplasts to the ER for glycerolipid assembly [40]. Our lipid profiling revealed that PC is mostly composed of polyunsaturated fatty acids such as 36:6-, 36:5-, 36:4, 36:3-, 34:3-, and 34:2-PC, and overexpression of *BnATS1* increased PC with polyunsaturated acyl groups. The ratio of polyunsaturated to saturated fatty acids of PC in *BnATS1*-OE plants (4.7–5.5:1) was substantially higher than that of WT plants (3.9:1). It was reported that plant species with a higher content of unsaturated fatty acids exhibit more tolerance to cold stress than those species containing a higher level of saturated fatty acids [41]. *Brassica napus* plants frequently experience cold stress under the growth conditions in the field during winter. Increased PC with polyunsaturated acyl groups in *BnATS1*-OE plants may contribute to enhanced tolerance to cold stress, thus promoting plant growth under low-temperature conditions.

However, increased PC in *BnATS1*-OE plants may not be resulting directly from increased BnATS1 expression, as PA produced in the prokaryotic pathway may be not involved in PC synthesis [2,3]. In agreement with these alterations, overexpression of *BnATS1* promotes the expression of genes such as *KAR*, *ATS2*, *PEAMT*, *AAPT1*, and *LPCAT* involved in de novo fatty acid synthesis, PC assembly, and acyl editing. In PC synthesis from the CDP-choline pathway, phosphoethanlamine is methylated to phosphocholine by PEAMT, and then is activated to CDP-choline [1,42]. The phosphocholine group of CDP-choline is further transferred to DAG by AAPT, producing PC [1,43]. LPCAT is involved in acyl editing through re-esterification of lyso-PC [10]. Our data showed that PC was largely increased in *BnATS1*-OE, which agrees with the increased expression of multiple genes in the lipid anabolic pathway. These results suggest that chloroplast-localized BnATS1 provides a “pull” force to enhance fatty acid synthesis and a “push” effect on glycerolipid assembly.

## 4. Materials and Methods

### 4.1. Plant Materials and Growth Conditions

The seeds of canola cultivar (cv.) Westar (*Brassica napus* L.) were germinated in pots containing soil. Two-week-old seedlings with comparable size were transferred to pots (one plant per pot) and grown with regular watering in field conditions from late autumn through the spring season in Wuhan, China. The temperature was approximately 7–10 °C (night)/15–20 °C (day) in late autumn and 0–3 °C (night)/7–12 °C (day) in winter in Wuhan, China.

### 4.2. Gene Cloning, Vector Construction and Plant Transformation

A cDNA pool was synthesized from mRNA extracted from leaves of cv. Westar (*Brassica napus*) plants by reverse transcription using a TransScript cDNA Synthesis SuperMix Kit according to the manufacturer’s instructions (TansGene Biotech, Beijing, China). The full-length *BnATS1* cDNA was amplified from cDNA pools by PCR using the primers *BnATS1*-OE-F 5′-TCTAGAATGACTCTCACGTTTTCCTC-3′ (forward) and *BnATS1*-OE-R 5′-GAGCTCCTAATTCCAAGGTTGTGACA-3′ (reverse), and then ligated into the expression vector pBI121 after digestion with *Xba*I and *Sac*I under the control of the 35S promoter. The construct containing *BnATS1* was confirmed by sequencing and then introduced into *Agrobacterium tumefaciens* strain GV3101, which was used to infect cv. Westar hypocotyls in tissue culture to get regeneration plants based on the methods described previously [44]. The transgenic plants were verified by PCR using the pBI121 vector sequence specific primer 5′-GATGGTTAGAGAGGCTTACGCA-3′ and *BnATS1* specific primer *BnATS1*-OE-R (Supplementary Table S1).

### 4.3. RNA Extraction and Real-Time PCR

Total RNA was isolated from various tissues of cv. Westar plants using Transzol reagent (TransGen Biotech, Beijing, China). RNA extracts were treated with DNaseI to remove contaminating DNA and used as a template to synthesize the first-strand cDNA by reverse transcription using a TransScript cDNA Synthesis SuperMix Kit according to the manufacturer’s instructions (TransGen Biotech, Beijing, China). *β-Actin* from *Brassica napus* was used as an internal standard. Real-time PCR was performed as described previously [45]. The primers used are listed in Supplementary Table S1.

### 4.4. BnATS1 Protein Expression and Activity Assay

The full-length *BnATS1* coding sequence (CDS) omitting the stop codon was amplified by PCR using primers *BnATS1*-PF and *BnATS1*-PR (Supplementary Table S1) and then was cloned into the pET28a vector at the cutting sites of *EcoR*I and *Xho*I. After sequence confirmation, the construct was introduced into the *E. coli* Rosetta (DE3) strain (TransGen Biotech, Beijing, China) and cultured in LB liquid medium until the OD_600_ reached 0.6 for protein expression by the induction with 0.5 mM isopropyl β-D-thiogalactopyranoside (IPTG) for 20 h at 16 °C. Cells were lysed by ultrasonification in cold lysis buffer (50 mM Tris-HCl, pH 7.5, 120 mM NaCl, 1 mM DTT, 0.1% Triton-100, 10% glycerol), and followed by centrifugation at 10,000 *g* at 4 °C for 20 min. BnATS1 activity was assayed in 100 μL of reaction mixtures containing 250 μM HEPES buffer (pH 7.4), 8 μM 16:0-CoA, 600 μM G3P-Ca, 5 mg/mL BSA, and 20 μg crude proteins incubated at 25°C for 5 min. The reaction was stopped by addition of 300 μL of chloroform: methanol (1:2, *v*/*v*). Resultant lipids were separated on thin layer chromatography (TLC) plates using a developing solvent (chloroform: methanol: acetic acid: H_2_O = 85: 15: 10: 3.5, *v*/*v*) and were visualized with iodine vapor [34]. The spots corresponding to LPA were quantified by the Image J software (1.48, National Institutes of Health, USA).

### 4.5. Subcellular Localization

The full-length *BnATS1* CDS was amplified by PCR using primers *BnATS1*-GFP-F 5′-GGTACCATGACTCTCACGTTTTCCTC-3′ (forward) and *BnATS1*-GFP-R 5′-TCTAGAATTCCAAGGTTGTGACAAAG-3′ (reverse) and cloned into the pCAMBIA1301s vector after digestion with *Kpn*I and *Xba*I, which results in in-frame C-terminal fusion to GFP. The construct was introduced into *Agrobacterium tumefaciens* GV3101. The transformant was grown overnight in liquid LB media, and then centrifuged at 4000 rpm for 10 min. The cells were resuspended with the solution containing 10 mM MgCl_2_ and 1 mM acetosyringone, and used for infiltrating leaves of 4-week-old tobacco (*Nicotiana benthamiana*) plants for transient expression driven by the 35S promoter. After infection for 2 to 3 days, BnATS1-GFP was visualized using a confocal laser scanning microscope (Leica, Biberach, Germany).

### 4.6. Lipid Extraction and Analysis

Total lipids were extracted according to the method described previously [46]. Briefly, leaf discs (0.5 g per sample) were sampled from 40-day-old plants (cv. Westar) and immediately immersed in 75°C isopropanol (5 mL) containing 0.01% butylated hydroxytoluene (BHT) for 15 min to arrest enzymatic activity. After cooling to room temperature (25–28 °C), the sovlent (3.5 mL) of chloroform/water (2.5:1, *v*/*v*) was added to the sample and incubated for 1 h at room temperature by shaking. The extracts were transferred to clean glass tubes, and then the remaining leaves were re-extracted with 5 mL chloroform/methanol (2:1, *v*/*v*) containing 0.01% BHT at room temperature for several times until leaf tissues became bleached. The extracts were combined and washed twice with 1 M KCl and once with water, and dried under a stream of nitrogen gas, and then dissolved in a defined volume of chloroform. Lipids were quantitatively profiled by electron-spray ionization tandem mass spectrometry (ESI-MS/MS) based on the approach described previously [46].

## Supplementary Materials

The following are available online at https://www.mdpi.com/1422-0067/21/15/5325/s1, Figure S1: Glycerolipid synthesis in two parallel pathways, Table S1: Primers Used in This Study.
