# Supplementary material for: Plastid Glycerol-3-phosphate Acyltransferase Enhanced Plant Growth and Prokaryotic Glycerolipid Synthesis in Brassica napus
Source: Int J Mol Sci. 2020 Jul 27;21(15):5325. doi: 10.3390/ijms21155325 (PMC7432870; doi:10.3390/ijms21155325)
Supplement: Supplementary file 1 [file ijms-21-05325-s001.pdf]

Supplementary

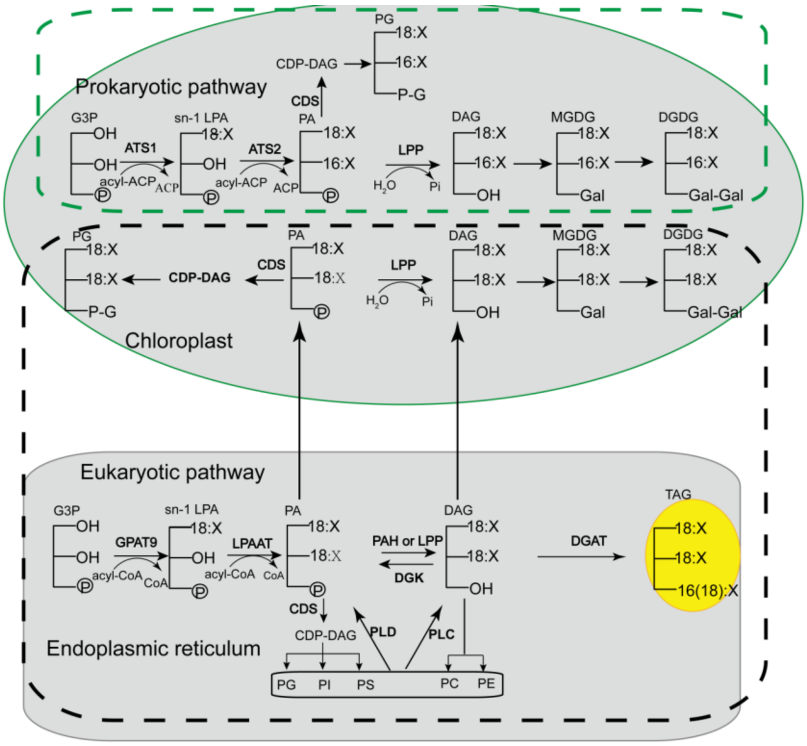

Supplementary Figure S1. Glycerolipid synthesis in two parallel pathways. Glycerolipids are assembled via the prokaryotic pathway in the chloroplasts or the eukaryotic pathway in the endoplasmic reticulum (ER). Glycerolipids originated from the prokaryotic pathway feature with a 16-carbon (C16) acyl moiety at the sn-2 position of the glycerol backbone, whereas those from the eukaryotic pathway have a 18-carbon (C18) acyl at the sn-2 position due to differing substrate selectivity of acyltransferases between the two pathways in plants. In addition, glycerolipids originated from the eukaryotic pathway can enter the chloroplasts, in the form of PA or presumably DAG produced from phospholipids, for the synthesis of thylakoid lipids such as PG, MGDG, and DGDG. Acyl-ACP, acyl–acyl carrier protein; acyl-CoA, acyl-coenzyme A; ATS, plastid-localized glycerol-3-phosphate acyltransferase; CDP-DAG, cytidinediphosphate diacylglycerol; CDS, cytidinediphosphate diacylglycerol synthase; DAG, diacylglycerol; DGAT, diacylglycerol acyltransferase; DGDG, digalactosyldiacylglycerol; DGK, diacylglycerol kinase; G3P, glycerol-3-phosphate; GPAT, glycerol-3-phosphate acyltransferase; LPA, lysophosphatidic acid; LPAAT, lysophosphatidic acid acyltransferase; LPP, lipid phosphate phosphatase; MGDG, monogalactosyldiacylglycerol; PA, phosphatidic acid; PAH, phosphatidic acid phosphohydrolase; PC, phosphatidylcholine; PE, phosphatidylethanolamine; PG, phosphatidylglycerol; PI, phosphatidylinositol; PLC, phospholipase C; PLD, phospholipase D; PS, phosphatidylserine; TAG, triacylglycerol.

Supplementary Table 1. Primers used in this study

Primer name	Primer sequence (5′ - 3′)	Gene locus
BnATS1-OE-F	TCTAGAATGACTCTCACGTTTTCCTC	BnaA08g06960D
BnATS1-OE-R	GAGCTCCTAATTCCAAGGTTGTGACA	
BnATS1-PF	GAATTCATGACTCTCACGTTTTCCTC	BnaA08g06960D
BnATS1-PR	CTCGAGCTAATTCCAAGGTTGTGACA	
BnATS1-GFP-F	GGTACCATGACTCTCACGTTTTCCTC	BnaA08g06960D
BnATS1-GFP-R	TCTAGAATTCCAAGGTTGTGACAAAG	
RT-*Bnactin*-F	TGTTCCCTGGAATTGCTGACCGTA	BnaC02g00690D
RT-*Bnactin*-R	TGCGACCACCTTGATCTTCATGCT	
BnATS1-RT-F	GGTTTCACGGTACTGGACTATC	BnaA08g06960D
BnATS1-RT-R	GCTTGGCTGTATGCTTCTTTC	
BnATS2-RT-F	AGTGGTGCCAATAACGCTGA	BnaC07g42750D
BnATS2-RT-R	TTCTGGCCTCGTCACAAAGA	
BnPEAMT-RT-F	TCCTGAAACGGGAACTGGAC	BnaC06g23520D
BnPEAMT-RT-R	TTTGAGTTCCACCCACCAACA	
BnAAPT1-RT-F	CTTGGTTGTACTTGGAACTGG	BnaC08g40270D
BnAAPT1-RT-R	GCGTTTGCAAGTGCAAAGGG	
BnLPCAT-RT-F	GGTTCCTGCGAAGCCCTCTCGGC	BnaA06g08020D
BnLPCAT-RT-R	GAGAGAAACAAGAACCGGACC	
BnKAR-RT-F	CTGGTGTTATTGGGTTCTCC	BnaC05g21140D
BnKAR-RT-R	CGTCCTAATGGGATTGTTCC	
BnPGPS-RT-F	GGTAGAGAGATTACAATGTC	BnaC03g22010D
BnPGPS-RT-R	CCGGCTTGCGAGAAGTATGGT	
